# Photoacoustic Signal Enhancement: Towards Utilization of Low Energy Laser Diodes in Real-Time Photoacoustic Imaging

**DOI:** 10.3390/s18103498

**Published:** 2018-10-17

**Authors:** Rayyan Manwar, Matin Hosseinzadeh, Ali Hariri, Karl Kratkiewicz, Shahryar Noei, Mohammad R. N. Avanaki

**Affiliations:** 1 Department of Biomedical Engineering, Wayne State University, 818 W. Hancock, Detroit, MI 48201, USA; r.manwar@wayne.edu (R.M.); karl.kratkiewicz@wayne.edu (K.K.); 2 Department of Electrical Engineering, Sharif University of Technology, Tehran 11365-11155, Iran; matinhz@gmail.com (M.H.); Sh.noei69@gmail.com (S.N.); 3 Department of Nano Engineering, University of California, San Diego, CA 92093, USA; haririali92@gmail.com; 4 Department of Dermatology, Wayne State University School of Medicine, Detroit, MI 48201, USA; 5 Barbara Ann Karmanos Cancer Institute, Detroit, MI 48201, USA

**Keywords:** photoacoustic imaging, signal enhancement, low-energy laser diodes

## Abstract

In practice, photoacoustic (PA) waves generated with cost-effective and low-energy laser diodes, are weak and almost buried in noise. Reconstruction of an artifact-free PA image from noisy measurements requires an effective denoising technique. Averaging is widely used to increase the signal-to-noise ratio (SNR) of PA signals; however, it is time consuming and in the case of very low SNR signals, hundreds to thousands of data acquisition epochs are needed. In this study, we explored the feasibility of using an adaptive and time-efficient filtering method to improve the SNR of PA signals. Our results show that the proposed method increases the SNR of PA signals more efficiently and with much fewer acquisitions, compared to common averaging techniques. Consequently, PA imaging is conducted considerably faster.

## 1. Introduction

Photoacoustic (PA) imaging, also referred to as optoacoustic (OA) imaging, is a fast-developing technique that utilizes the PA effect to noninvasively visualize the concentration of compartments in biological tissues [1,2,3,4,5,6,7]. PA imaging is used in a large number of preclinical and clinical applications. In PA imaging, a short-pulsed laser is used to illuminate a biological tissue. A portion of light is absorbed by absorbing compartments in the tissue, such as oxy- and deoxy hemoglobin [6,8]. The absorbed energy is converted to heat, causing thermoelastic expansion, resulting in ultrasonic waves, which when detected by ultrasound transducers, will be converted to PA signals [5,9]. A PA signal is usually contaminated with background noise, comprised of a combination of electronic noise and system thermal noise [10,11]. On the other hand, because tissues are highly scattering and the PA waves generated in a tissue undergo many attenuation events before arriving at transducers, there will always be some other components of noise present in the PA signal [12]. We call the combination of these unwanted signals, the “measurement noise”, known to complicate the interpretation of resulting PA signals and deteriorate PA image quality [13].

Low-energy pulse laser diodes (LE-PLD) are used as a light source in cost-effective PA imaging systems [14,15,16]. The PA signals generated by LE-PLD usually have a very low signal-to-noise ratio (SNR) and the quality of reconstructed images is not satisfactory [17]. To enhance the SNR, different varieties of spectral filtering and averaging techniques have been applied [18]. For instance, in [16], a short-lag spatial coherence (SLSC) beam forming algorithm has been investigated to improve the contrast of PA images. Due to the overlapping spectrum of noise and bandwidth of PA signals, after filtering the degraded PA signal results in low PA image quality. Several groups have studied other signal enhancement techniques. The most common techniques include the empirical mode decomposition (EMD) method, wavelet-based methods, Wiener deconvolution, and principle component analysis (PCA). In the EMD method [19,20,21], finding an appropriate criterion for reconstruction of the PA image based on intrinsic mode function components, is still a challenge. An optimum threshold value in wavelet transformation is also difficult to find. One proposed solution is to make the threshold process parametric. The heuristic methods proposed for tuning threshold values are usually complicated and require expensive computation [22,23,24]. Wiener deconvolution methods have been used to reduce noise by equalizing phase and amplitude characteristics of the transducer response function. Although Wiener filtering is effective, it heavily depends on adequate estimation of correlation functions of the signal and noise [25,26]. SNR enhancement using the PCA method is insignificant because it assumes that the ratio of PA energy to the total energy of detected signals is more than 75%, which is not always the case [27]. PA signal analysis indicates that the signal and noise around it (the samples collected before or after the PA peak) are uncorrelated. It also shows that after pulse-pulse laser fluctuation compensation, there is only a slight PA peak signal fluctuation in different acquisitions [28]. Therefore, averaging different sampling epochs will improve the SNR of the signal. Although, direct signal averaging within the frames will lead to losing high-frequency components that primarily reflect on small-size structures in the PA image. Cross-correlation operations can be used to determine the time-shift between every two PA waveforms. Most of the previous denoising methods require prior knowledge about the signal or noise properties. For example, in the wavelet algorithm, the choice of wavelet mother function and threshold value, depend on characteristics of the signal and noise. Digital spectral filtering requires a great deal of knowledge about the spectrum of the signal and noise. Several adaptive filtering methods have also been studied for PA signal enhancement. These methods, with almost no prior knowledge about the signal or noise and with fewer parameters to tune, could yield significant noise reduction [29,30,31]. Moreover, the reduced sensitivity of adaptive methods to tuning parameters is advantageous. The adaptive method proposed in [32], is based on spline interpolation of PA signals. Even though the authors demonstrated a significant improvement in the quality of images, the use of spline for smoothing PA signals and calculating the cross-correlation of signals for fine-alignment before averaging, is an intensive computational task, which limits the feasibility and application of the method to systems where real-time imaging is required. Another PA adaptive denoising algorithm has been proposed in Reference [33], where spatial resolution is doubled by utilizing an adaptive noise canceller (ANC). With an ANC, there is a need to define a reference signal, which can be defined as a signal that is correlated with the measurement noise. In an LE-PLD based PA system, the signal is typically buried in white noise and finding an appropriate reference signal that significantly correlates with the noise may not be easy or feasible. Without a reference signal the ANC switches itself off and has no effect on the noisy input signal.

In this paper, we present an adaptive and fast-filtering method to denoise and enhance the PA signal SNR. However, unlike a conventional ANC, it does not require a reference input or prior knowledge about characteristics of the signal. In fact, the reference signal is basically a time shifted version of the primary input signal. A detailed theoretical background of the proposed algorithm and filter modeling is presented. Later, we explain the experimental setup and associated methodologies. Finally, the performance of the proposed algorithm is evaluated through phantom experiments where the PA signal amplitude enhancement, PA signal peak shift, and execution time are investigated.

## 2. Adaptive Denoising Filter Design

A conventional adaptive noise cancellation filter can be described as follows. If x(n) is the noisy measured signal consisting of the original signal s(n), contaminated by noise $v_{0}\left(n\right)$:

(1) $$\;x\left(n\right)=s\left(n\right)+v_{0}\left(n\right)\;$$

v(n) is the reference signal and $\hat{v}\left(n\right)$ is the output of the adaptive filter. Additionally, minimizing the mean squared of the error (MSE) leads to:

(2) $$\min\left(E\left[{\hat{s}}^{2}\right]\right)=\min\left(E\left[s^{2}\right]+E\left[{\left(v-\hat{v}\right)}^{2}\right]+2E\left[s\left(v-\hat{v}\right)\right]\right)\;$$

where E[.] stands for mathematical expectation and $\hat{s}$ represents expected output signal. If the signal and noise are uncorrelated i.e., $E\left[sv\right]=E\left[s\hat{v}\right]=0$ then Equation (2) simplifies to:

(3) $$\min\left(E\left[{\hat{s}}^{2}\right]\right)=E\left[s^{2}\right]+\min\left(E\left[\left(v-\hat{v}\right)\right]\right)\;$$

Minimizing MSE cancels out correlative terms. Since the signal in the output remains constant, minimizing the total output power maximizes the output SNR.

**Figure 1:**
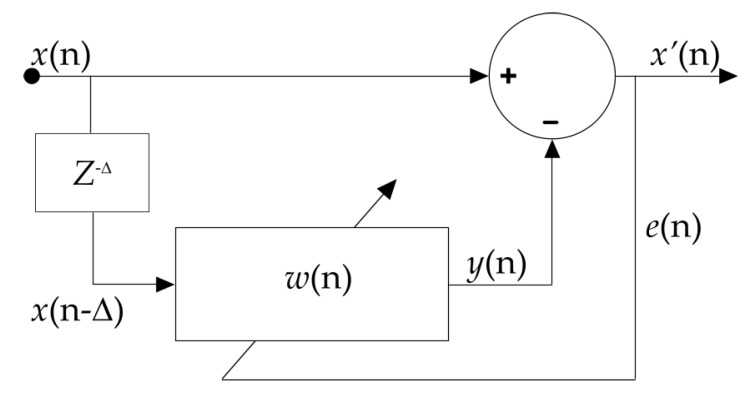
Schematic diagram of the proposed filter.

In the proposed algorithm, input of the predictor (adaptive filter) is a delayed version of x(n), i.e., x(n−Δ), where Δ is equal to or greater than one. The main role of Δ is to remove the correlative part in the signal. The algorithm works best when either the signal or noise is broadband and the other is narrowband. This is because a broadband signal has a wide power spectral density (PSD). Hence, the inverse Fourier transformation of PSD, or autocorrelation function is narrow, which results in a decreased correlation between the signal and its delayed version. The only criterion that needs to be ensured is that the introduced delay should be set at a greater value than the decorrelation-time of the broadband signal and less than the decorrelation time of the narrowband signal. A PA signal is a narrowband signal when compared to the broadband Gaussian measurement noise [34]. The block diagram of the proposed algorithm is shown in Figure 1. In this figure, x(n) is the noisy measured signal, y(n) is the output of the adaptive filter, and Δ is the decorrelation delay parameter. The value of delta is selected empirically, i.e., the algorithm is performed on a subset of data using different Δ values and the delta value corresponding to the best SNR was selected.

It should be noted that delta is the only hyper parameter that needs to be tuned just once in each imaging experiment. The coefficients of the predictor with *N* taps, can be denoted as a vector $w^{T}=\left[w_{0},w_{1},\ldots ,w_{N-1}\right]$ where T represents transpose operation. The output can be described as follows:

(4) $$y\left(n\right)=w^{T}\cdot x^{T}\left(n-\Delta \right)\;$$

(5) $$x^{T}\left(n-\Delta \right)=\left[x\left(n-\Delta \right)\cdot x\left(n-\Delta -1\right)\ldots \cdot x\left(n-\Delta -N+1\right)\right]\;$$

The predictor output in Equation (4) is an estimate of noise where the signal is narrowband compared to the noise. The LMS algorithm is utilized to estimate components of $w^{T}$, where the prediction error is e(n)=x(n)−y(n). The LMS algorithm updates coefficients such that $E\left[x\prime^{2}\left(n\right)\right]$ is minimized. Coefficients of the adaptive filter are calculated using Equation (6):

(6) w(n+1)=w(n)+μ⋅e(n)⋅x(n−Δ) 

where μ is the step size that controls convergence of the adaptation process. Global convergence is guaranteed if μ is bounded as $0<\mu <2/\lambda_{\max}$, where $\lambda_{\max}$ is the greatest eigenvalue of the input autocorrelation matrix, i.e., $R=E\left[x\left(n-\Delta \right)\cdot x^{T}\left(n-\Delta \right)\right]$.

The main drawback of the LMS algorithm is that it is sensitive to the scaling of its input. This makes it challenging to choose a step size that guarantees stability of the algorithm. The normalized least mean squares (NLMS) algorithm is a variant of the LMS algorithm that solves this problem by normalizing the power of the reference signal. The adaptation rule of the NLMS method is given in Equation (7):

(7) $$w\left(n+1\right)=w\left(n\right)+\frac{\mu \cdot e\left(n\right)\cdot x\left(n-\Delta \right)}{x^{T}\left(n-\Delta \right)\cdot x\left(n-\Delta \right)}\;$$

Convergence of this adaptation process is guaranteed if the step size *μ*, is bounded in 0 < *μ* < 2. Performance of the NLMS method depends on the characteristics of the input signal, the value of decorrelation delay, and length of the adaptive filter.

## 3. Materials and Methods

**Figure 2:**
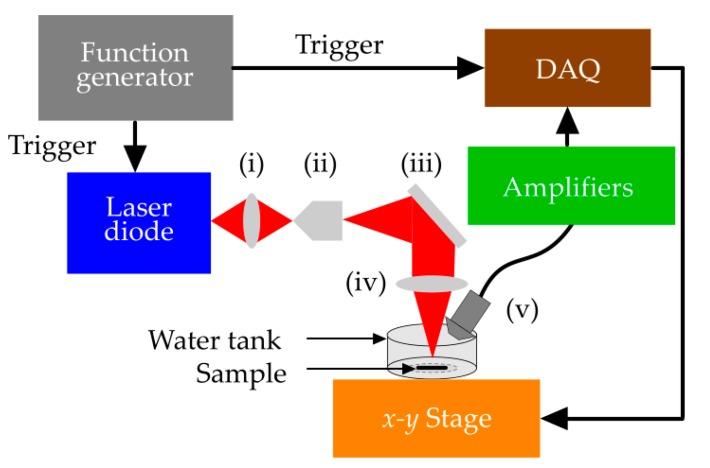
Schematic of a low-cost photoacoustic microscopy setup., (i) convex lenses (focal length of 100 mm), (ii) objective lens, (iii) mirror, (iv) convex lens (focal length 60 mm), and (v) unfocused ultrasound transducer.

Figure 2 shows a schematic diagram of the experimental setup of a low-cost PA microscope that is used in this study to evaluate performance of the proposed algorithm.

A pulsed laser diode (PLD) (905D1S03X, Laser Component Co., Bedford, NH, USA) with a peak power of 6 W, pulse width of 55 ns operating at a maximum repetition rate of 20 kHz, and a wavelength of 905 nm has been used in the experiment with 125 nJ laser energy. For focusing, the diverging beam out of the PLD was allowed to pass through a convex lens (AC254-100-A1, Thorlabs, Newton, NJ, USA) with the focal length of 100 mm and the diameter of 50 mm. An objective lens (40×; Newport cooperation, Irvine, CA, USA) with 0.65 numeric aperture (NA) and magnification of 40 was used to make the beam spherical. The beam was then reflected by a mirror and focused onto the sample by means of a 60 mm focal length convex lens (AC254-060-A1, Thorlabs) with a diameter of 50 mm. The optical design of experimental setup and its parameters were optimized based on ZEMAX simulations [5]. The incident energy on the sample was measured to be 30 nJ. Assuming the focal spot to be 0.5–2 mm below the surface of the sample, the maximum surface optical influence is estimated to be below 2 µJ/cm^2^, which is far below the maximum permissible exposure (MPE) at this wavelength according to the American National Standards Institute (ANSI) safety standard [35]. A motor (Applied Motion Products Inc., Watsonville, CA, USA) driven *x-y* mechanical stage (ASI LX-4000; Applied Scientific Instrumentation, Inc., Eugene, OR, USA) was used for scanning with the precision of 1 µm. The PA signal was detected by a stationery unfocussed ultrasonic transducer (V312-SU immersion transducers; Olympus) with an active element diameter of 6 mm and a central frequency of 10 MHz. We chose 10 MHz to balance between adequate resolution and having sufficient penetration depth [36]. Characteristics of the transducer do not affect the efficiency of the proposed filter. However, choosing a larger bandwidth transducer makes finding the decorrelation delay parameter easier. The signal was amplified by a cascade of three low noise amplifiers (500 series ZFL Mini Circuits RF/Microwave Components; Brooklyn, NY, USA) with a total gain of 69.54 dB. Data acquisition was performed using an FPGA-based National Instrument (NI) data acquisition (DAQ) unit with a sampling rate of 200 MS/s. The function generator (ATTEN ATF20B, ATTEN.EU, Helmond, Netherlands) was set to generate 20 k square pulses/s with 50% duty cycle to trigger both the DAQ and laser diode. LabVIEW was used for signal visualization and analysis. Analysis of the autocorrelation of 1000 recordings (from different samples) using low-cost PA microscopy revealed that most of the measured signals have only a slight or no autocorrelation with more than four samples of time lag. Therefore, the decorrelation delay (Δ) was set to 5.

## 4. Results and Discussion

**Figure 3:**
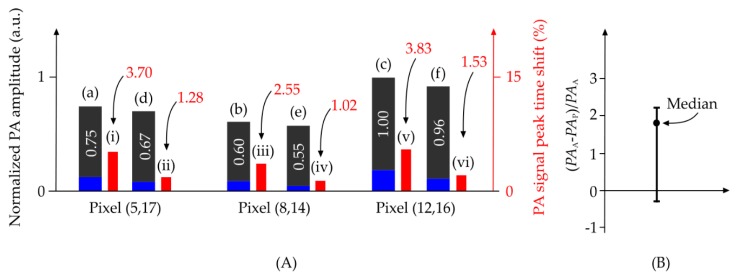
Performance comparison between averaging and the proposed denoising method on three photoacoustic (PA) signals. (**A**) (a–c) Denoised PA signals using averaging 150 signals for three-pixel locations, (d–f) denoised PA signals using the proposed method for the same pixels when 15 acquisition epochs were used, (i, iii, v) PA signal peak time-shift when averaging, and (ii, iv, vi) PA signal peak time-shift when the proposed algorithm was used. (**B**) Percentage of difference between the PA amplitude obtained from averaging (PA_A_) and PA amplitude obtained from the proposed algorithm (PA_P_) divided by the PA amplitude obtained from averaging, for all pixels in the image. The dark grey and blue color bars in A indicate the peak amplitude of the PA signal and noise at the pixel location respectively. The red color bars represent the PA peak time-shift percentage.

To evaluate the proposed denoising method we used a trimmed homogenous black tape strip made of vinyl as the image target. Height and width of our PA images were equally set to 20 pixels. First, we reconstructed the photoacoustic microscopy (PAM) image by averaging 250 acquisitions for each pixel location. This image was used as the reference image. We chose three PA sample signals belonging to three different pixel locations on the image and denoised them using both the common averaging technique (150 acquisitions) as well as our algorithm (15 acquisitions). The results are shown in Figure 3A. Figure 3B shows the percentage of difference between the PA amplitude obtained from averaging and PA amplitude obtained from the proposed algorithm, divided by the PA amplitude obtained from averaging, for all pixels in the image. These results show that the proposed method produces similar results to averaging with a much smaller number of epochs, i.e., a reduced computational complexity.

**Figure 4:**
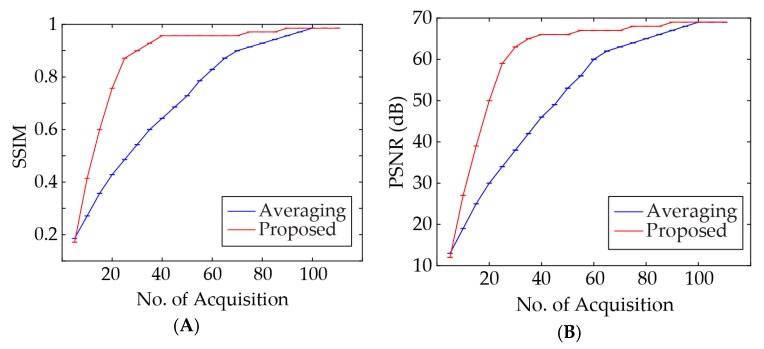
(**A**) Structural similarity index measure (SSIM) and (**B**) peak signal to noise ratio (PSNR) versus the number of acquisitions for the proposed method and conventional averaging method.

The averaging method leads to a PA signal peak shift in the temporal domain. The time shift caused by averaging and the proposed algorithm are compared. As seen in Figure 3A, the time shift caused by the proposed algorithm when 15 acquisition epochs are used, is almost one third of that caused by conventional averaging when 150 acquisition epochs are used. Figure 4A,B depict the variation of structural similarity index measure (SSIM) and peak signal to noise ratio (PSNR) with different numbers of acquisitions for the conventional averaging method and the proposed algorithm.

**Figure 5:**
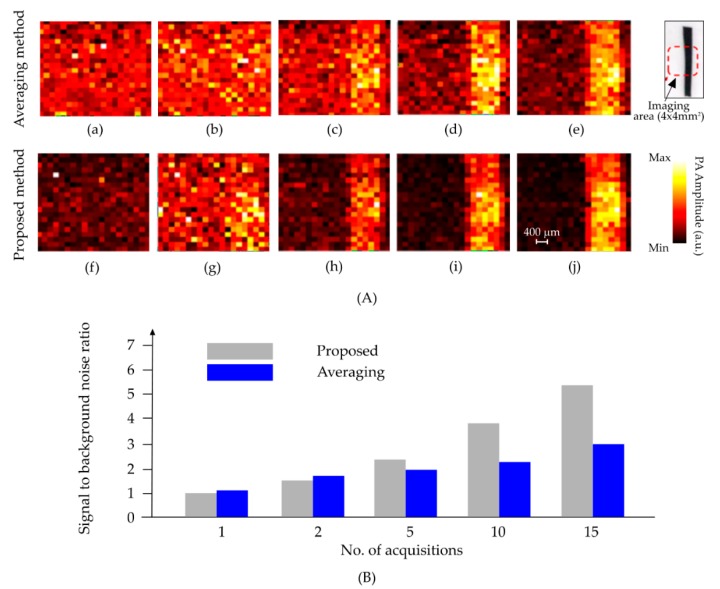
(**A**) Comparison between the averaging method and proposed method for 1 (a, f), 2 (b, g), 5 (c, h), 10 (d, i), and 15 (e, j) signal acquisitions. (**B**) Signal to background ratio comparison between the averaging method and proposed method for 1, 2, 5, 10, and 15 signal acquisitions.

It can be inferred that performance of the adaptive algorithm surpasses the averaging method for a wide range of acquisition numbers. For further comparison between the proposed method and conventional averaging technique, images were reconstructed with different numbers of acquisitions. The results are shown in Figure 5 and the corresponding statistics are listed in Table 1.

**Figure 6:**
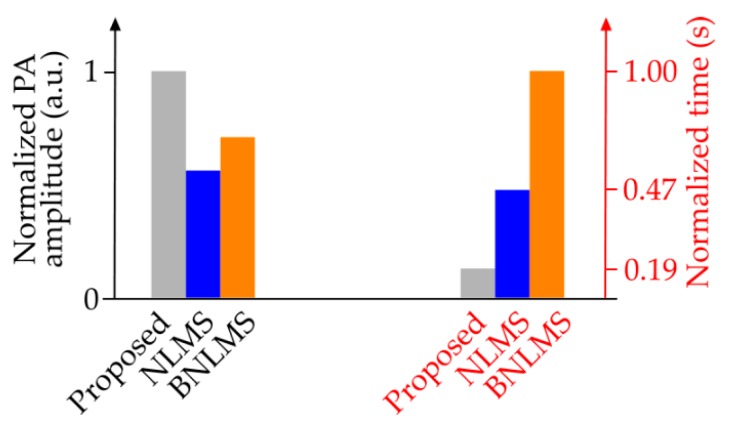
Performance comparison between normalized least mean square (NLMS), block normalized least mean square (BNLMS) filtering, and proposed methods in terms of signal amplitude enhancement and computational complexity.

We also compared the results of the proposed method with those from two other established adaptive filtering methods, i.e., normalized least mean square (NLMS) and block normalized least mean square (BNLMS), in terms of both PA signal amplitude enhancement and computational complexity. The results are shown in Figure 6.

To generate a PAM image with an acceptable SNR (30 dB), at least 150 signal acquisitions are required to be averaged for each pixel location (when a PAM system is used as shown in Figure 2). Such acquisition and averaging will take about 0.6 ms for each pixel location (with a laser repetition rate of 500 kHz). Hence, a PAM image with the size of 200 × 200 pixels will take about 12 s (including the arming time in the DAQ and post-processing), which does not allow real-time PA imaging. Using the proposed method (and 10 acquisitions) each pixel in the image will take only 1.2 s.

## 5. Conclusions

An adaptive noise cancellation based denoising algorithm has been presented and experimentally compared to the conventional averaging technique to verify the superior performance of this algorithm. Even though convergence of the proposed algorithm requires the acquired signal to be sufficiently long, it has a self-learning ability. This algorithm is based on the assumption of having at least one uncorrelated component between the actual input and its delayed version. However, the proposed algorithm does not require any prior knowledge of the signal nor the noise, as compared to the digital filtering method, where sufficient prior knowledge is essential to accurately determine the type of filter and cut-off frequencies. Moreover, this algorithm stands out among adaptive filtering methods by not needing a reference signal and requiring less computational complexity. Due to using a reduced number of epochs in averaging, our algorithm creates a smaller PA peak time-shift and signal-broadening. A PAM image with the size of 200 × 200 pixels using the proposed method took about 1 s, which allows real-time PA microscopy. An ex-vivo study based on phantom tissue will be performed in the future.

## Figures and Tables

**Table 1:** Comparison between averaging and the proposed method for different numbers of acquisitions.

No. of Signal Acquisitions	Method	PSNR	SSIM
1	Averaging	15.83	0.22054
	Proposed	15.67	0.13695
2	Averaging	19.58	0.33235
	Proposed	22.39	0.37588
5	Averaging	24.73	0.53189
	Proposed	32.08	0.73874
10	Averaging	28.85	067040
	Proposed	38.68	0.93019
15	Averaging	31.54	0.75843
	Proposed	40.10	0.93797
